# Application of ultrasound in a congenital cystic adenomatoid malformation in an adult: A case report

**DOI:** 10.1097/MD.0000000000023505

**Published:** 2020-12-04

**Authors:** Wen Xu, Qing Wen, Lijing Zha, Chunmei Liu, Pintong Huang

**Affiliations:** Department of Ultrasound, Second Affiliated Hospital of Zhejiang University, School of Medicine, Hangzhou, China.

**Keywords:** adult, congenital cystic adenomatoid malformation, contrast enhanced ultrasound, lung

## Abstract

**Introduction:**

Congenital cystic adenomatoid malformation (CCAM) is a rare developmental lung abnormality, that typically manifests in neonates and infants but rarely in adults. Ultrasound is an important method of diagnosing CCAM in neonates and infants; however, few articles have reported the value of transthoracic lung ultrasound in the diagnosis of CCAM in adults.

**Patient concerns:**

We present a case of a 34-year-old woman with a cavitary lesion in her left lower lobe, that suggested chronic inflammation.

**Diagnosis:**

The patient underwent ultrasound examination and contrast-enhanced ultrasound-guided transthoracic core biopsy; histology suggested the diagnosis of lung hamartoma. Surgical resection of the lesion followed by histopathological analysis confirmed the diagnosis of CCAM.

**Interventions:**

The patient underwent transthoracic core biopsy under contrast-enhanced ultrasound guidance. A left lower lobectomy was then performed subsequently.

**Outcomes:**

The patient had a smooth recovery and remained asymptomatic during the 12-months of postoperative follow-up.

**Conclusion:**

We report a rare case of CCAM to suggest that transthoracic ultrasound combined with contrast-enhanced ultrasound is a safe and effective method of diagnosing the subpleural lung malformations in adults, thereby avoiding multiple radiation exposures and associated complications.

## 1 Introduction

Congenital cystic adenomatoid malformation (CCAM) is a rare developmental lung abnormality, that typically manifests in neonates and infants. It rarely remains asymptomatic until adulthood. Recurrent pulmonary infection is a common complication of CCAM.^[1]^ The preoperative diagnosis is challenging, especially when it appears as a solid mass or a single uniloculated cyst. Ultrasound is an important method of diagnosing CCAM in neonates and infants.^[2]^ However, few articles have been reported the application of transthoracic lung ultrasound in the diagnosis of CCAM in adults. In this article, we report a case of a 34-year-old woman with a cavitary lesion in her left lower lobe, that suggested chronic inflammation. The patient underwent ultrasound examination and contrast-enhanced ultrasound-guided transthoracic core biopsy; histology revealed lung hamartoma. Surgical resection of the lesion followed by histopathological examination confirmed the diagnosis of CCAM.

## 2 Case report

**Figure 1:**
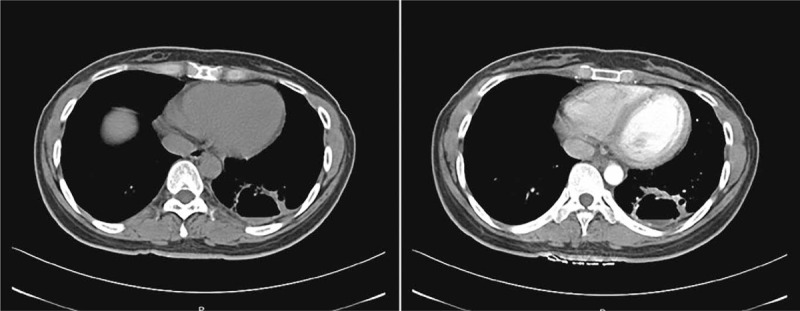
An enhanced thoracic CT scan showed a cavitary lesion with fluid level located in the left lower lobe of the lung, ∼60 mm ^∗^ 45 mm ^∗^ 36 mm in size. The wall of the lesion was thick and obviously enhanced after the enhancement. The pleura around the cavity was thickened and adherent. Imaging finding was indicative of pulmonary chronic inflammation.

**Figure 2:**
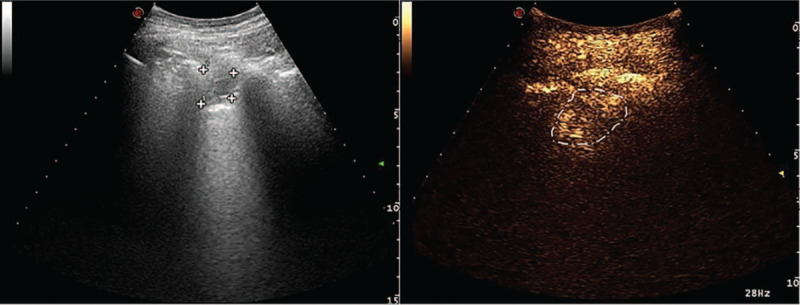
Transthoracic lung ultrasound showed hypoechoic subpleural lesion in her left lower lobe. The lesion was inhomogeneous with clear margins, ∼24 mm ^∗^ 22 mm in size. The contrast enhanced ultrasound presented a small amount of perfusion in periphery, but no perfusion in the center of the lesion. On ultrasound and contrast enhanced ultrasound imaging, the lesion was indicative of a chronic inflammatory lesion.

**Figure 3:**
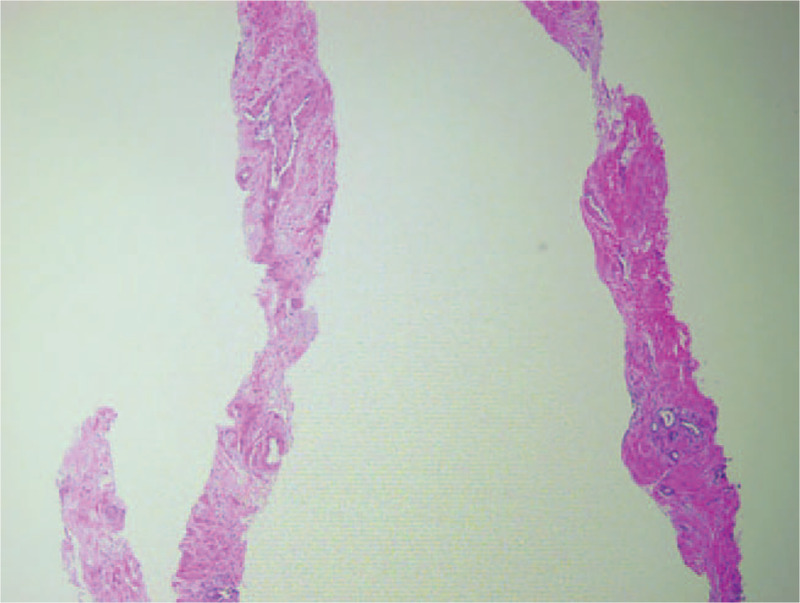
Histopathological analysis of lung biopsy specimen revealed hyperplastic fibrous tissue, irregular blood vessels, skeletal muscle, smooth muscle, and bronchus, considered as pulmonary hamartoma.

**Figure 4:**
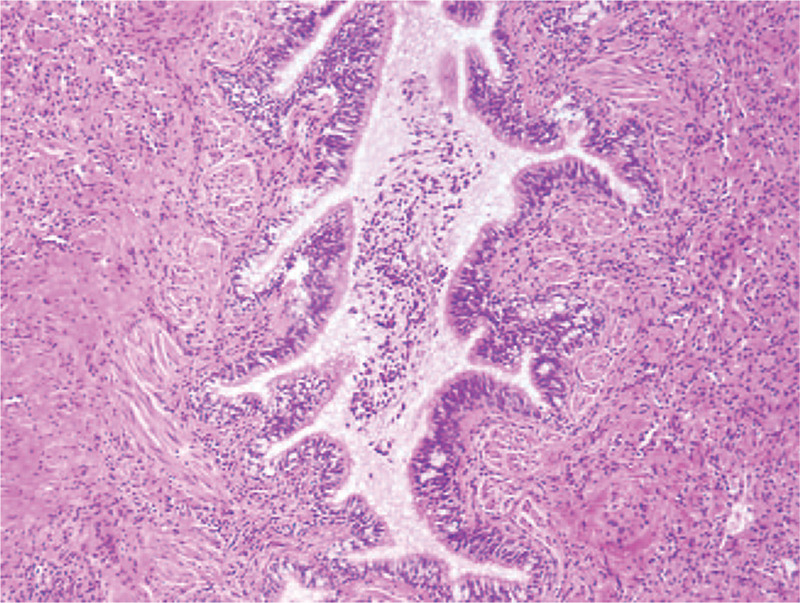
Histopathological analysis of lung resection specimen indicated chronic inflammation of the lower left lung tissue, with dysplasia glandular ducts and cystic cavities formation. The largest diameter of the cystic cavity was >2 cm. The smallest one was ∼2 mm to 5 mm. The cyst wall was covered with ciliated columnar epithelium.

A 34-year-old woman was admitted to the department of respiratory medicine with left back pain for more than 6 months, aggravating for 5 days. No specific medical and family history was included. Routine laboratory tests were unremarkable. An enhanced thoracic computed tomography (CT) scan showed a cavitary lesion with fluid level and thickened wall in the left lower lobe of the lung, ∼60 mm ∗ 45 mm ∗ 36 mm in size. The thickness of the thickest wall was ∼5.6 mm and obviously enhanced after the enhancement (Fig. 1). The pleura around the cavity was thickened and adherent. On CT imaging, the lesion was indicative of pulmonary chronic inflammation. Transthoracic lung ultrasound showed a hypoechoic subpleural lesion in her left lower lobe. The lesion was inhomogeneous with clear margins, ∼24 mm ∗ 22 mm in size. The contrast-enhanced ultrasound (CEUS) presented a small amount of perfusion in periphery, but no perfusion in the center of the lesion (Fig. 2). At ultrasound and contrast enhanced ultrasound imaging, the lesion was indicative of a chronic inflammatory lesion, consistent with the CT findings. Core biopsy of the solid components of the lesion guided by CEUS was performed; histology revealed the diagnosis of hamartoma (Fig. 3). The left lower lobectomy was performed 3 weeks later and showed the pleura adhesions and the partial aplasia interlobar fissure. The cyst was tough in texture and located in the posterior internal basal segment of the left lower lobe, ∼40 mm in size. Lymph nodes were found in mediastinum and hilum. Histopathological analysis of the lung resection specimen indicated the chronic inflammation of the lower left lung tissue, with dysplasia glandular ducts and cystic cavities formation. The largest cystic cavity was >2 cm in diameter. The smallest one was ∼2 to 5 mm. The cyst wall was covered with ciliated columnar epithelium. Immunohistochemistry revealed CK20 +, CK7 ++, CK5/6 +, P63 +, P53 −, PD-L1 −, PDL1-NC −, TTF-1 +, NapsinA +, SMA +, S-100 −, and Desmin ++. The histology and immunohistochemistry confirmed the diagnosis of mixed type of CCAM (Fig. 4). The patient had a smooth recovery period and remained asymptomatic during the 12-months of postoperative follow-up period.

All the invasive operations and treatments were approved by the patient and her families, and written informed consent was provided. Patient provided informed consent for publication of this case.

## 3 Discussion

CCAM is a rare developmental, hamartomatous lung abnormality with unknown etiology.^[3]^ CCAM is a type of adenomatoid lung hamartoma^[3]^ that is easily misdiagnosed as pulmonary hamartoma. Histopathological analysis of lung biopsy specimen in this case revealed hyperplastic fibrous tissue, irregular blood vessels, skeletal muscle, smooth muscle, and bronchus, that was suggestive of pulmonary hamartoma.

In 1977, Stocker grossly classified CCAM into three types primarily according to the morphologic spectrum,^[4]^ type I: single or multiple large cysts (>2 cm); type II: multiple small cysts (<2 cm); type III: a solid mass. In 2002, CCAM was renamed as congenital pulmonary airway malformation (CPAM) and was expanded into five types, adding the types 0: solid tissue involving both lungs due to congenital acinar dysplasia, and type IV: a large peripheral cyst. The types 0 and type IV are almost exclusively diagnosed in infants.^[5]^ In this case, the histological results showed that the largest diameter of the cystic cavity was >2 cm and the smallest one was approximately 2 to 5 mm in size, suggesting a diagnosis of mixed-type CCAM.

The preoperative diagnosis in an adult is challenging, especially when it appears as a solid mass or single uniloculated cyst, since the congenital abnormality may be radiologically mistaken for something more common in adult group than in the pediatric population. In this case, the cavitary lesion was misdiagnosed as chronic inflammation in both CT and US imaging. Ultrasound is an important method of diagnosing CCAM in neonates and infants.^[2,6]^ The sonographic findings vary from a single large cystic lesion to multiple hypoechoic lesions, and/or consolidation.^[7]^ However, few articles have reported the value of ultrasound for diagnosing CCAM in adults. Several articles indicated that CEUS could dynamically display the microvascular features within the pulmonary lesion, delineate the nonenhanced areas, and sensitively detect large vessels inside the lesions, which can be very useful for the biopsy.^[8]^ It was also reported that biopsy of the enhanced regions within the lesion could significantly improve the diagnosis rate to 98.3%.^[9]^ In this case, conventional ultrasound showed an inhomogeneous hypoechoic subpleural lesion in left lower lobe. After CEUS, biopsy was designed to target the enhanced areas in periphery. Adequate specimen was obtained, and no side effects occurred in this case. Thus, the transthoracic lung ultrasound combined with CEUS is a safe and effective method of diagnosing the subpleural lesion of lung malformations in adults. However, it showed that the size of the lesion measured by ultrasound was smaller than CT, possibly because of the different positioning, the scanning section, the limited intercostal space, as well as the surrounding gas interference. In addition, the value of ultrasound decreases as the lesion's location depth increases and the successful biopsy rate also depends on the operator's experience.

Surgical removal of CCAMs is recommended by some experts because of the possibility of increased risk of malignancy, while others propose a conservative follow-up with serial chest CT scans. Bronchioloaveolar carcinoma, pulmonary rhabdomyosarcoma and pulmonary blastoma have been reported in association with CCAM in several studies.^[10–12]^ In the current case report, the prognosis after surgery remained good and there was no evidence of recurrence or metastasis after surgery. The patient recovered well and remained asymptomatic during the 12-months of postoperative follow-up period.

## Author contributions

**Data curation:** Qing Wen, Chunmei Liu.

**Methodology:** Lijing Zha.

**Writing – original draft**: Xu Wen.

**Writing – review & editing**: Huang Pintong
